# The Influence of Diet and Physical Activity on Periodontal Health: A Narrative Review

**DOI:** 10.3390/dj13050200

**Published:** 2025-04-30

**Authors:** Giuseppe Balice, Michele Paolantonio, Giovanna Murmura, Matteo Serroni, Stefania Di Gregorio, Beatrice Femminella

**Affiliations:** Department of Innovative Technologies in Medicine and Dentistry, “G. D’Annunzio” University, 66100 Chieti-Pescara, Italy; giuseppe.balice@phd.unich.it (G.B.); giovanna.murmura@unich.it (G.M.); matteo.serroni@phd.unich.it (M.S.); stefania.digregorio@unich.it (S.D.G.); beatrice.femminella@unich.it (B.F.)

**Keywords:** dietary pattern, periodontal health, periodontitis, obesity, physical activity, micronutrients, Mediterranean diet, plant-based diet

## Abstract

Periodontal diseases, including gingivitis and periodontitis, are chronic inflammatory conditions that compromise the supporting structures of the teeth, often leading to tooth loss and contributing to systemic comorbidities. Increasing evidence underscores the critical role of modifiable lifestyle factors, particularly diet and physical activity, in influencing periodontal health. This narrative review critically evaluates the current body of literature regarding the impact of dietary constituents and physical activity on the periodontium, with a focus on the molecular mechanisms, key biomarkers, and clinical implications. It aims to provide a deeper understanding of the complex interactions between nutrition, exercise, and periodontal health with potential implications for clinical management and preventive strategies.

## 1. Introduction

Periodontal diseases are among the most prevalent chronic conditions globally. Between 2011 and 2020, it was estimated that approximately 62% of adults were affected by periodontitis, with 23.6% experiencing the severe form of the disease [1]. Severe periodontitis represents the primary etiological factor in tooth loss among adults, leading to compromised masticatory function, heightened risk of malnutrition, and a significant reduction in overall quality of life [2]. Furthermore, factors such as the growing global population, changing risk factors, and improved tooth retention are expected to amplify the socio-economic burden of periodontitis, which currently contributes to 3.5 million years lived with disability, an annual loss of 54 billion USD in productivity, and a substantial portion of the 442 billion USD per year associated with the overall cost of oral diseases [3]. 

Periodontitis is a chronic, multifactorial inflammatory disease characterized by a dysbiotic shift in the oral microbiota, which initiates a cascade of host-mediated immune responses, ultimately resulting in the progressive destruction of the periodontal apparatus [4]. This pathological process is primarily triggered by the accumulation of bacterial biofilms on the tooth surface, which disrupt the ecological balance and homeostasis of the periodontium, a functional unit comprising the gingiva, periodontal ligament, cementum, and alveolar bone. The subsequent inflammatory response is mediated by complex interactions between microbial virulence factors and host immune mechanisms, leading to connective tissue breakdown, alveolar bone resorption, and, if left untreated, eventual tooth loss [5,6]. 

While microbial dysbiosis and host genetic susceptibility are widely recognized as key drivers of periodontal pathogenesis, there is growing evidence highlighting the role of modifiable lifestyle factors—particularly diet and physical activity (PA)—modulating periodontal health.

Diet affects immune responses, oxidative stress, and inflammatory processes within periodontal tissues [7], while regular PA may modulate these factors, ultimately influencing the progression of periodontal disease [8]. This narrative review aims to integrate the current body of evidence on the influence of diet and PA on periodontal health.

## 2. Dietary Factors Affecting Periodontal Health

### 2.1. Micronutrients and the Periodontium

#### 2.1.1. Vitamin C and Collagen Synthesis 

##### Biological Rationale 

Vitamin C, a potent antioxidant, cannot be synthesized by the human body due to the absence of the enzyme L-gulono-gamma-lactone oxidase [9,10,11], making it an essential nutrient that must be obtained through diet. Vitamin C serves multiple critical functions, including its essential role in collagen synthesis and stabilizing its tertiary structure, which is crucial for maintaining the integrity of connective tissues, such as the periodontium [12,13]. Additionally, ascorbate is vital for the biosynthesis of catecholamines (dopamine, norepinephrine, and epinephrine), acting as a cofactor for dopamine β-hydroxylase, thereby supporting the synthesis of dopamine and norepinephrine. During sepsis, β-adrenergic receptor downregulation and impaired adrenal hormone synthesis are observed, while ascorbate deficiency reduces norepinephrine levels, particularly in the adrenal glands. Ascorbate also enhances adrenergic receptor activity, and the brain and adrenal glands are the tissues with the highest ascorbate concentrations, emphasizing its crucial role in catecholamine function. Supplementing ascorbate during sepsis may aid in catecholamine synthesis and receptor activation [14].

**Figure 1:**
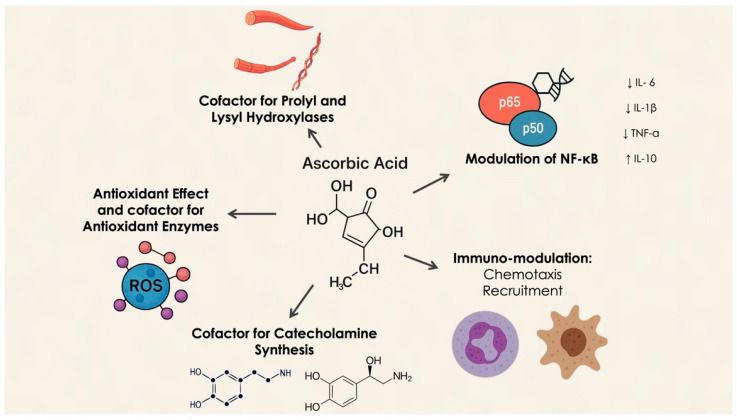
Molecular mechanism of vitamin C activity.

As an antioxidant, vitamin C protects periodontal health by neutralizing reactive oxygen species (ROS) produced during inflammation. Excessive ROS can exacerbate periodontal damage by disrupting cellular structures and interfering with signaling pathways that regulate inflammatory responses [15]. Furthermore, vitamin C supports immune function by enhancing phagocyte activity and promoting T-lymphocyte proliferation, both of which are vital for defending against periodontal pathogens [16]. Neutrophils utilize Sodium-Dependent Vitamin C Transporter 2 (SVCT2) to absorb vitamin C, and during oxidative bursts, they increase intracellular ascorbate levels via non-specific uptake of dehydroascorbate (DHA), which is subsequently reduced to ascorbate. This high accumulation of vitamin C helps protect neutrophils from oxidative damage, scavenging ROS and regenerating antioxidants like glutathione and vitamin E. Depletion of vitamin C during phagocytosis or stimulation with soluble agents impairs immune responses by disrupting the balance between ROS generation and antioxidant defenses, leading to the activation of pro-inflammatory pathways such as Nuclear Factor kappa-light-chain-enhancer of activated B cells (NF-κB) [17] (Figure 1).

Moreover, vitamin C modulates neutrophil chemotaxis, enhancing their migration to infection sites in response to chemical signals. It also improves neutrophil phagocytosis and microbial killing by boosting ROS generation. Studies indicate that supplementation with vitamin C enhances these immune functions, with ascorbate deficiency resulting in impaired neutrophil apoptosis and clearance [18].

An inadequate intake of vitamin C can ultimately result in the development of scurvy. The hallmark clinical feature of this condition is vascular fragility, which manifests through a range of hemorrhagic symptoms, including petechiae, ecchymoses, hematuria, epistaxis, subperiosteal bleeding, and intramuscular hemorrhages—collectively referred to as “scurvy siderosis” [19]. 

Oral manifestations are particularly significant, characterized by swollen, bleeding gums and the loosening of teeth. Histologically, scurvy is marked by dilated subepithelial blood vessels within the gingival tissues, which are prone to rupture, leading to hemorrhage in the surrounding connective tissue. Poor oral hygiene exacerbates gingival inflammation, and in advanced cases, this can progress to periodontal tissue damage, alveolar bone resorption, and ultimately tooth loss [19,20]. In pediatric populations, vitamin C deficiency is also associated with odontoblastic dysfunction, resulting in malformed teeth due to impaired dentinogenesis. This dental dysplasia is characterized by the formation of amorphous dentin lacking dentinal tubules, reflecting defective collagen synthesis that compromises the structure and function of the periodontal ligament. Scurvy in infants commonly arises during the transition from breastfeeding to nutritionally inadequate formula feeding, particularly when formulas lack sufficient vitamin C. In these cases, the gingiva may exhibit hyperplastic overgrowth, encasing the clinical crowns of the teeth and contributing to tooth mobility and loss as the supporting periodontal structures deteriorate [21,22].

Although scurvy is uncommon in developed nations, it remains a concern among specific at-risk populations. These include elderly individuals, those with substance use disorders, individuals following restrictive or unbalanced diets, and persons with psychiatric disorders. Additional high-risk groups include smokers, individuals exposed to secondhand smoke, infants and young children primarily fed cow’s milk, patients with end-stage renal disease undergoing chronic hemodialysis, and individuals with malabsorptive gastrointestinal conditions. Moreover, the body’s demand for vitamin C increases in response to physiological stressors such as illness, surgical recovery, exposure to certain drugs or toxins, and environmental or occupational stress. For instance, studies have shown that South African miners working underground require daily vitamin C intakes of 200–250 mg to maintain normal plasma levels. Furthermore, lifestyle factors such as smoking and the use of oral contraceptives are known to significantly reduce circulating levels of ascorbic acid, increasing the risk of deficiency [23].

##### Scientific Evidence

A systematic review by Tada et al. found an adverse correlation between plasma levels, vitamin C intake, and incidence of periodontal disease [24]. The review indicated that individuals with lower dietary intake or serum levels of vitamin C exhibited more rapid progression of periodontal disease compared to controls. The authors concluded that adequate vitamin C intake plays a protective role in reducing the risk of periodontitis. This was confirmed in another more recent systematic review by Cengel et al. that highlighted the detrimental effects of vitamin C deficiency on periodontal health, demonstrating that vitamin C supplementation significantly reduces gingival inflammation and clinical attachment loss (CAL) [25]. Deficiencies in Vitamin C are strongly associated with both gingivitis and periodontitis, as impaired collagen formation compromises the gingival tissues, making them more vulnerable to bacterial invasion [13]. Furthermore, a pilot study by Munday et al. [26] examined vitamin C levels in periodontal patients and found that vitamin C deficiency (serum levels < 11 μmol/L) was associated with diminished healing capacity and increased gingival inflammation. In addition, low serum vitamin C levels were correlated with more severe forms of periodontitis and higher levels of C-reactive protein. Additionally, multiple studies suggest the protective effect of Vitamin C: antioxidant properties are critical in mitigating oxidative stress within the periodontium, thereby contributing to periodontal tissue protection [27]. In a study by Vogel et al., the impact of high-dose vitamin C on polymorphonuclear leukocyte chemotaxis in the development of experimental gingivitis was evaluated. After four months of daily supplementation with vitamin C (500 mg, three times per day), a significant increase in plasma ascorbate concentrations was observed. However, this did not result in enhanced host defense during the 4-week period of experimental gingivitis [28,29]. 

However, it is also important to consider the method of intake. A study by Eberhardt et al. [30] demonstrated that the antioxidant effect of vitamin C when obtained from food sources is more effective than dietary supplementation. The researchers assessed antioxidant capacity through the total oxyradical scavenging capacity (TOSC) assay. Their results indicated that 1 g of apples with the peel exhibited a total antioxidant activity of approximately 83.35 TOSC, expressed in millimoles of vitamin C equivalents. This implies that 100 g of apples have an antioxidant potential comparable to 1500 mg of vitamin C. Considering that the average concentration of vitamin C in 100 g of fresh apples with skin is around 5.7 mg, and that this amount (i.e., 0.057 mg per gram) corresponds to merely 0.32 TOSC, the data strongly suggest that the vast majority of the antioxidant activity in apples derives from phytochemicals rather than vitamin C alone. 

The presence and function of vitamin C in the gingival crevicular fluid (GCF) have been a subject of increasing scientific interest due to its potential implications in periodontal health. A pivotal study by Meyle and Kapitza [31] using high-performance liquid chromatography demonstrated that the mean concentration of ascorbic acid in GCF (207 pmol/L) is significantly higher than that observed in serum (72 pmol/L; *p* < 0.001). 

These findings suggest that GCF may represent a locally enriched microenvironment for ascorbate, possibly regulated independently from systemic circulation. The elevated local concentration of ascorbic acid may result from active epithelial secretion or cellular release, potentially from gingival fibroblasts or infiltrating immune cells. This is consistent with the known preferential accumulation of ascorbic acid in leukocytes and platelets, indicating a tissue-specific transport mechanism and suggesting a role in localized antioxidant defense and immune regulation [16]. 

Despite these physiological properties, previous clinical trials have failed to demonstrate significant therapeutic benefits from systemic megadoses of vitamin C, particularly in enhancing polymorphonuclear neutrophils chemotaxis or improving periodontal treatment outcomes [28,32]. One plausible explanation lies in the already elevated baseline levels of vitamin C in GCF, which may render systemic supplementation redundant due to a saturation threshold at the tissue level. Recent experimental data support the efficacy of local vitamin C delivery in modulating periodontal disease. In a rat model of ligature-induced periodontitis, Aytekin et al. [33] reported that intragingival injection of ascorbic acid significantly reduced the expression of 8-hydroxy-2′-deoxyguanosine (8-OHdG)—a biomarker of oxidative DNA damage—and Matrix Metalloproteinase (MMP)-8, a key collagenase associated with tissue degradation. 

These findings suggest that ascorbic acid exerts both antioxidant and anti-catabolic effects within the periodontal milieu. In addition to biochemical markers, vitamin C-treated animals exhibited improved clinical parameters, including significant reductions in CAL and alveolar bone loss (ABL) compared to untreated controls. These beneficial effects occurred without significant changes in systemic Tumor Necrosis Factor-alpha (TNF-α) levels, likely reflecting the localized action of the treatment and the limited diffusion of inflammatory mediators from small tissue compartments into systemic circulation. 

This supports the concept of compartmentalized inflammation in periodontal disease. Mechanistically, vitamin C appears to function via a dual pathway: it attenuates oxidative stress and concurrently downregulates tissue-destructive enzymatic activity, potentially by modulating gene expression related to inflammation and extracellular matrix remodeling [31]. 

Interestingly, while systemic administration of vitamin C has shown inconsistent outcomes in clinical trials [33,34], local application has emerge as a more effective and targeted approach. This discrepancy may stem from the challenge of achieving sufficient concentrations at the periodontal site through systemic delivery alone without inducing adverse effects [35]. As supported by multiple studies [33,34,35,36], topical vitamin C administration enables therapeutic efficacy with lower doses and minimal systemic exposure. Collectively, these findings emphasize the clinical potential of locally delivered ascorbic acid in the management of periodontal disease. Its capacity to modulate oxidative stress, inflammatory signaling, and connective tissue metabolism suggests that vitamin C may serve not only as an adjunctive therapy but also as a biologically active agent capable of restoring periodontal homeostasis. 

#### 2.1.2. Vitamin D and Inflammatory Modulation 

##### Biological Rationale 

**Figure 2:**
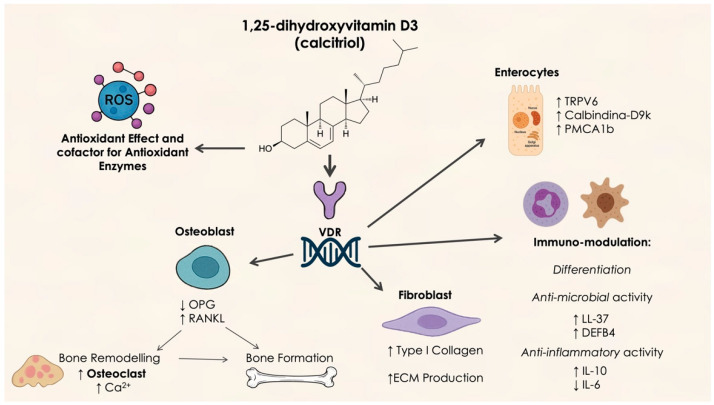
Molecular mechanism of vitamin D activity. *OPG:* osteoprotegerin; *RANKL*: Receptor Activator of Nuclear Factor κB Ligand; *TRPV6*: Transient Receptor Potential Vanilloid 6; *PMCA 1b*: plasma membrane Ca^2^⁺-ATPase; *LL-37*: Antimicrobial peptide Leu-Leu 37; *DEFB4*: Defensin Beta 4.

Vitamin D is a crucial nutrient and precursor hormone that is involved in various biochemical processes within the body [37]. This lipid-soluble vitamin is primarily obtained through dietary sources in the forms of vitamins D2 and D3, as well as through sunlight exposure. Both forms of vitamin D are converted to 25-hydroxyvitamin D (25(OH)D) in the liver, then further transformed into the active metabolite 1,25-dihydroxyvitamin D3 (1,25(OH)2D3) in the kidneys. 25(OH)D, being the predominant form found in circulation, serves as the key marker for assessing vitamin D levels and storage in the body [38]. A deficiency in vitamin D has become a significant public health concern globally. Research has shown that insufficient vitamin D levels are associated with an increased risk of periodontitis [39]. This link may be due to the role of vitamin D in regulating calcium and bone metabolism as well as immune system function. Consequently, it is hypothesized that vitamin D may influence the onset and progression of periodontitis through its effects on bone homeostasis and immune responses [40] (Figure 2).

##### Scientific Evidence 

Vitamin D plays a critical role in immune modulation and bone metabolism, both of which are essential for periodontal health. It helps regulate the inflammatory response to periodontal pathogens and enhances calcium absorption, thereby supporting the integrity of alveolar bone. Pinto et al. [41] reviewed the effects of vitamin D on periodontal disease and concluded that individuals with low vitamin D levels exhibited more severe periodontal disease, suggesting its potential as a therapeutic adjunct. A review by Liu et al. [42] corroborated the association between vitamin D deficiency and increased severity of periodontal disease, emphasizing its role in mitigating inflammatory responses.

Machado et al. [43] conducted a descriptive analysis on the role of vitamin D supplementation in non-surgical periodontal therapy (NSPT), but the study lacked sufficient quantitative data to support its effectiveness in adjunctive non-surgical treatments like scaling and root planning (SRP). Conversely, a more recent systematic review and meta-analysis by Liang et al. [40] confirmed that SRP combined with vitamin D supplementation significantly reduced CAL compared to SRP alone, aligning with Machado et al.’s findings. This review also noted lower serum vitamin D levels in periodontal patients compared to healthy individuals and concluded that SRP combined with vitamin D was effective in reducing CAL but did not lead to significant improvements in probing depth (PPD) or bleeding on probing (BoP).

The vitamin D receptor (VDR) plays a central role in immune responses and is present on the plasma membranes of various immune cells, including monocytes, macrophages, and dendritic cells. Calcitriol, the active form of vitamin D, binds to VDRs on immune cells, enhancing macrophage chemotactic and phagocytic activities by stimulating the expression of 1-α-hydroxylase in monocytes and macrophages. This leads to the autocrine production of calcitriol, which increases lysosomal enzyme activity and phagocytosis [44]. Calcitriol also binds to VDRs on junctional and gingival epithelial cells, contributing to epithelial defense against pathogens. Activation of Toll-like receptors (TLR2/1 and TLR4) induces 1-α-hydroxylase expression, further enhancing calcitriol production and amplifying its effects. The relationship between VDR gene variants and periodontitis has garnered considerable attention [45,46,47]. Unlike hereditary 1,25(OH)2D3-resistant rickets, a rare monogenic disorder caused by a mutation in the VDR gene, single nucleotide polymorphisms (SNPs) are more common and represent subtle variations in genetic sequences. Specific VDR genotypes have been correlated with periodontitis, resulting in increased ABL, CAL, and tooth loss [47,48,49]. Recent meta-analyses have indicated that the FokI gene polymorphism for VDR is significantly associated with heightened susceptibility to periodontitis, while associating BsmI polymorphism with FokI has been linked to an increased risk of developing periodontitis in the general population [50,51]. 

In conclusion, the involvement of vitamin D in periodontal health is multifaceted, encompassing its regulatory effects on alveolar bone homeostasis and mineralization, enhancement of epithelial barrier function against microbial insult, and modulation of the immune response. Furthermore, vitamin D exhibits direct antimicrobial effects and downregulates pro-inflammatory signaling pathways within the periodontal microenvironment [44]. 

Recent findings from Yıldırım et al. [52] have demonstrated that serum vitamin D concentrations are inversely correlated with MMP-9 levels in patients with periodontal diseases. Although vitamin D levels were not directly assessed in gingival crevicular fluid (GCF), this study underscores a significant indirect role for systemic vitamin D status in modulating local periodontal inflammation. The inverse association with MMP-9—a key enzyme involved in extracellular matrix degradation—highlights the capacity of vitamin D to exert protective effects by attenuating tissue destruction in both gingivitis and periodontitis. 

Emerging evidence underscores the crucial role of vitamin D-binding protein (DBP)—the principal carrier of vitamin D metabolites—in modulating the local bioactivity of vitamin D within periodontal tissues. Li et al. [53] demonstrated that DBP is not only systemically present but also locally expressed in both dental and periodontal compartments. Intriguingly, DBP levels were found to be higher in GCF than in plasma under healthy conditions, suggesting a tissue-derived source and a potential active role in local regulation [52]. DBP contributes to vitamin D bioavailability, enabling autocrine and paracrine signaling in periodontal cells such as gingival epithelial cells, fibroblasts of the periodontal ligament, and dental pulp cells. Beyond transport functions, DBP facilitates immune surveillance by enhancing neutrophil and monocyte chemotaxis via complement component C5a binding and by serving as a precursor to DBP-macrophage activating factor (DBP-MAF), which modulates macrophage responses to inflammation [53]. Additionally, DBP-MAF has been implicated in bone remodeling by influencing osteoclast activity, thereby suggesting a role in maintaining alveolar bone integrity. These insights support a broader conceptualization of vitamin D as a local immune and bone-modulatory agent in the periodontal niche. Furthermore, the diagnostic relevance of DBP in GCF is gaining traction. Chakravarthy et al. [54] evaluated serum and GCF levels of DBP in patients with chronic periodontitis and periodontally healthy controls. Their results revealed significantly elevated DBP levels in both serum and GCF among periodontitis patients. The authors concluded that DBP levels in GCF may serve as a valuable biomarker for monitoring periodontal tissue destruction and disease progression.

Together, these findings underscore the dual systemic and local roles of vitamin D and DBP in the regulation of periodontal immune responses, tissue integrity, and bone metabolism—reinforcing their potential utility as therapeutic targets and diagnostic biomarkers in periodontitis.

#### 2.1.3. Omega-3 Fatty Acids and Inflammation 

##### Biological Rationale

Omega-3 polyunsaturated fatty acids (ω-3 PUFAs), including docosahexaenoic acid (DHA; C22:6, n-3) and eicosapentaenoic acid (EPA; C20:5, n-3), have been recognized for their anti-inflammatory, protective, and therapeutic effects on conditions such as rheumatoid arthritis, ulcerative colitis, atherosclerosis, cancer, cardiovascular diseases, and periodontitis, as reported by Simopoulos [55]. It is well established that ω-3 fatty acids help reduce eicosanoids, including prostaglandin E2 and inflammatory cytokines, which act as key inflammatory mediators [56]. Serhan et al. [57] identified a novel class of lipid mediators, resolvins and protectins, which are enzymatically produced from ω-3 fatty acids. These mediators play a crucial role in reducing neutrophil infiltration and promoting monocyte recruitment. Given that the body’s ω-3 fatty acid reserves are limited, their levels in tissues are primarily determined by dietary intake [55]. ω-3 fatty acids can be converted into specialized pro-resolving mediators (SPMs), such as resolvins D and E, protectins, and neuroprotectins, which have a key role in managing and resolving inflammation [58]. In fact, several studies [59,60,61,62] have investigated the impact of EPA and DHA on oxidative stress, showing that these ω-3 fatty acids can reduce inflammation and lower oxidative stress levels.

##### Scientific Evidence

In a review by Miroult et al. [63], ω-3 fatty acids were shown to reduce the Gingival Index (GI), PPD, and CAL in patients with periodontitis treated with SRP. Ω-3s also modulate the immune response, potentially shifting the balance from pro-inflammatory to anti-inflammatory cytokine production, thereby reducing tissue destruction [64]. A randomized clinical trial by Deore et al. [65] observed a reduction in GI and Bleeding Index (BI), both clinical indicators of inflammation in periodontitis, in the patients that consumed a 300 mg gelatin capsule of ω-3 PUFAs (EPA and DHA) daily compared to the control group, in which no ω-3 PUFAs were administrated, without any substantial differences in plaque accumulation between groups. Additionally, in a six-week randomized controlled trial, Bartha et al. [66] evaluated the serum levels of PUFAs in individuals adhering to the Mediterranean diet (MD), exploring the associations between the intake of specific foods and their potential correlations with oral inflammation markers. The authors found that patients adhering to MD showed a decrease in ω-6 serum levels, resulting in a more favorable ω-6/ω-3 ratio and, at the same time, a reduction in periodontal bleeding. Another recent systematic review and meta-analysis by Van Ravensteijn et al. [67] concluded that adjunctive use of ω-3 fatty acids (2000 mg/day of EPA and/or DHA) to SRP resulted in 0.39 mm more PPD reduction and 0.41 mm in CAL gain compared to SRP alone. However, a study by Rosenstein et al. [68] compared clinical periodontal outcomes at 12 weeks in 30 patients with periodontitis who were administered either 3000 mg of fish oil daily (source of ω-3 PUFAs), 3000 mg of borage oil (source of ω-6 PUFAs and gamma-linolenic acid) daily, 1500 mg of fish oil and 1500 mg of borage oil daily, or a placebo. The results indicated that supplementation with borage oil, a source of ω-6 PUFAs, and gamma-linoleic acid may have positive effects on periodontal inflammation. Supplementation with ω-6 PUFAs seemed to produce more notable improvements than either ω-3 PUFA supplementation or the combination of the two lower-dose supplements. 

#### 2.1.4. Minerals and Periodontitis

##### Biological Rationale

Calcium, magnesium, iron, and zinc are essential micronutrients for the physiological maintenance of tissue homeostasis. Approximately 99% of the body’s total calcium is stored in bones and teeth as hydroxyapatite, with less than 1% present in soft tissues and body fluids. The extracellular calcium concentration in blood is tightly regulated between 2.2 and 2.6 mmol/L by three hormones: parathyroid hormone, 1,25-dihydroxycholecalciferol, and calcitonin. Intracellular calcium fluctuations trigger numerous biological responses, including changes in membrane permeability, neurotransmitter release, muscle contraction, enzyme activation, and gene transcription [69]. Low calcium intake, particularly in combination with insufficient vitamin D, can lead to low serum calcium levels, stimulating parathyroid hormone secretion. This, in turn, leads to osteoclastogenesis and bone resorption as a compensatory mechanism to prevent hypocalcemia. Magnesium functions as a cofactor or activator for more than 300 enzymatic reactions, notably those involved in ATP-dependent metabolic pathways. In parallel, iron is a vital trace mineral required for numerous metabolic activities in almost all forms of life, including humans. While its most well-known role is facilitating oxygen transport through hemoglobin, iron also plays an essential role as a cofactor in a wide range of enzymatic systems. A deficiency in iron availability can lead to compromised enzymatic efficiency and impaired metabolic function [70].

Zinc is a crucial micronutrient involved in a wide array of physiological processes. It contributes significantly to human growth and development, tissue repair, cellular proliferation and differentiation, gene regulation, membrane stability, and cytoskeletal structure. Moreover, zinc plays an important role in antioxidant defense mechanisms, immune system function, and overall metabolic regulation. Acting as a cofactor for more than 300 enzymes, it is integral to the metabolism of carbohydrates, lipids, proteins, and nucleic acids. Zinc is also essential for the intracellular interaction between tyrosine kinase and T-cell receptors (CD4 and CD8a), a process critical for the maturation and activation of T-lymphocytes. In biological systems, zinc is predominantly found in its divalent form (Zn^2^⁺) and is distributed across all body tissues and fluids [71].

##### Scientific Evidence

Several studies have evaluated the importance of calcium concentration in blood for periodontal health. In a 7-year study, men with low calcium intake (<1000 mg/day) experienced 30% more tooth loss due to ABL compared to those with higher calcium intake (≥1000 mg/day) [72]. Additionally, Nishida et al. [73] observed that low dietary calcium intake was associated with more severe periodontitis in young adults and middle-aged men. This was particularly evident when total serum calcium levels were combined with dietary intake. In fact, a recent study by Cao et al. [74] found significant differences in serum calcium levels among individuals with periodontitis and healthy controls, and they concluded that elevated serum calcium levels appear to be associated with a lower risk of periodontitis in adults, representing a preventive means for periodontitis [69,74].

Meisel et al. [75,76] found a significant relationship between magnesium status and periodontal health. Hypomagnesemia was present in 35% of participants, and those using magnesium-containing drugs showed less attachment loss and more remaining teeth compared to non-users. The study suggests that magnesium supplementation could improve periodontal health and prevent or delay tooth loss, particularly in middle-aged individuals. A case-control study from India reported improvements in serum magnesium levels and micronutrients after NSPT in patients with periodontitis, including those with controlled and uncontrolled diabetes [77]. Multiple studies have linked iron and periodontitis. Studies have shown that individuals with periodontitis tend to have lower erythrocyte counts and hemoglobin levels compared to healthy controls [78]. This is consistent with the idea that periodontitis may lead to anemia, potentially due to suppressed erythropoiesis caused by the systemic inflammatory response to periodontal pathogens [79]. Furthermore, improvements in hematological parameters, such as increased erythrocyte counts and hemoglobin, were observed up to 6 months after NSPT in periodontal patients [80]. Both periodontitis and iron deficiency anemia induce oxidative stress, leading to an imbalance between ROS and antioxidants such as superoxide dismutase (SOD). Moreover, patients with both periodontitis and iron deficiency anemia exhibit more severe periodontal symptoms, such as BoP, CAL, and deeper pocket depths [81]. However, a study by Pushparani and Nirmala [82] observed higher serum iron levels in patients with both type 2 diabetes and periodontitis compared to healthy controls, suggesting that the relationship between iron levels and periodontal disease may vary based on other factors. Similarly, Thomas et al. [83] did not find significant differences in iron levels between periodontitis patients and controls. A case-control study conducted by Enhos et al. [84] investigated the effects of NSPT on iron levels in Turkish female patients with and without iron deficiency anemia. Following treatment, significant increases in serum iron, ferritin, and hemoglobin levels were observed in the group with iron deficiency anemia, while total iron binding capacity decreased. In contrast, the group without iron deficiency anemia showed no significant changes in most hematological parameters.

While no adverse effects have been linked to the intake of naturally occurring zinc from food, problems may arise from zinc supplementation. Excessive zinc intake can suppress the immune response and interfere with lysyl oxidase, an enzyme crucial for collagen crosslinking and maintaining the structural integrity of connective tissues, which is essential for wound healing [85]. Zinc has also been shown to have an antagonistic effect on iron, with high iron intake potentially reducing zinc absorption and vice versa [86,87]. There is ongoing debate about the sensitivity of plasma and serum zinc concentrations as indicators of zinc status, as they represent only 1–2% of the body’s total zinc pool [85,88,89]. A recent study by Zhou et al. [90] revealed an association between serum zinc levels and periodontitis. The adjusted odds of periodontitis were 9% lower (odds ratio [OR]: 0.91; 95% confidence interval [CI]: 0.83–1.00) for nonsmokers and 14% lower for smokers; the authors concluded that serum zinc levels were associated with the risk of periodontitis, considering serum zinc as a variable associated with periodontitis.

## 3. Relationship Between Dietary Patterns and Periodontitis

**Figure 3:**
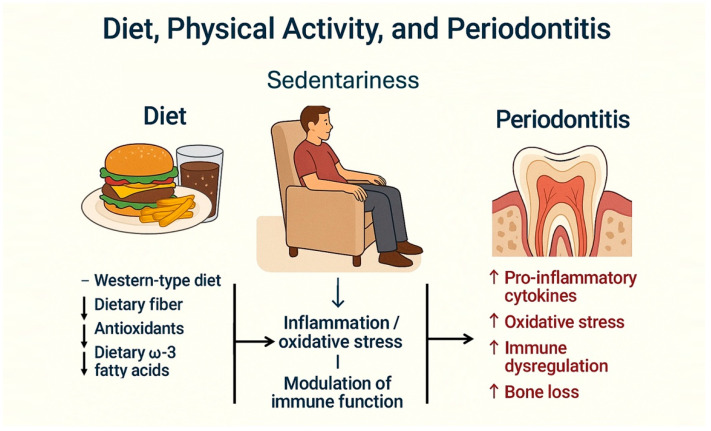
Interrelationship between Western-type diet, sedentary lifestyle, and periodontitis. A Western-type diet, typically low in dietary fiber, antioxidants, and omega-3 fatty acids, combined with a sedentary lifestyle, contributes to increased systemic inflammation and oxidative stress. These conditions impair immune regulation, facilitating the development and progression of periodontitis.

Several studies have assessed the relationship between diet type and periodontal disease [7,91]. It has been observed that diet can influence systemic inflammatory status and may act as a risk factor for several chronic systemic diseases [92]. A recent systematic literature review evaluated the effect of four dietary patterns on periodontal health [93] and concluded that various diets significantly affect periodontal conditions (Figure 3).

### 3.1. Western Diet

The Western diet (WD), typical of Western countries and rich in sugars, processed foods, fast foods, convenience products, snacks, and sugary soft drinks, yet deficient in fibers, vitamins, and minerals, is associated with increased body weight (BW), pathological changes in lipids and energy metabolism, and systemic inflammation. The WD is energy-dense, high in glycemic index, and leads to rapid blood glucose spikes, promoting excessive calorie intake and fat storage, thereby increasing weight [93,94]. Studies indicate that the WD contribute to elevated inflammation markers, directly or indirectly affecting the immune system [95,96]. Elevated cholesterol levels, often from animal fats and meat, are linked to inflammation and atherosclerosis [97]. Furthermore, oxidized Low-Density Lipoprotein cholesterol (oxLDL) can activate macrophages, contributing to atherosclerotic plaque formation and inflammation [98]. Saturated fatty acids (SFAs) from animal fats may induce immune cell stress and activate the NLRP3 inflammasome, promoting further inflammation [99]. Moreover, red meat and dairy products contribute to cardiovascular disease (CVD) risk through the conversion of L-carnitine into inflammatory molecules like trimethylamine N-oxide (TMAO) by the gut microbiota [100], with an increased risk of periodontitis [101].

### 3.2. Mediterranean Diet

In contrast to the WD, the MD, based on the dietary habits of Southern Italians and Greeks during the 1950s and 1960s [102], is rich in vegetables, fresh fruits, olive oil, and whole grains, with minimal red meat consumption, favoring fish. A review by Dinu et al. [103], which included 13 meta-analyses of observational studies and 16 randomized controlled trials (RCTs), involving over 12.8 million participants, suggests that adherence to the MD reduces the risk of chronic diseases and overall mortality. Strong evidence supports its role in reducing cardiovascular diseases, coronary heart disease, and myocardial infarction, with protective effects against atherosclerosis by reducing risk factors such as weight, Body Mass Index (BMI), waist circumference (WC), total cholesterol (TC), and increasing High-Density Lipoprotein (HDL) cholesterol. It also demonstrates a robust association with a reduced risk of diabetes, although its effect on metabolic syndrome was weaker. RCTs suggest improved glycemic control and reduced insulin resistance in individuals following the MD.

In the field of periodontology, a randomized controlled clinical trial by Bartha et al. [104] investigated the effect of the MD on clinical oral inflammation over 6 weeks. The study found that, despite the Plaque Index (PI) remaining constant between groups, patients following the MD showed improved inflammatory markers [BOP, GI, and Periodontal Inflamed Surface Area (PISA)] and better anthropometric parameters: BW, BMI, and WC. Laiola et al. [105] observed that, after 8 weeks, overweight and obese patients adhering to MD exhibited a significant reduction in the abundance of periodontopathogenic species such as *Porphyromonas gingivalis*, *Prevotella intermedia*, and *Treponema denticola* compared to the control group, which followed a diet rich in animal proteins. The authors concluded that increased adherence to the MD could reduce the abundance of periodontopathogenic bacteria in the saliva of overweight individuals with cardiometabolic risk without affecting their energy intake, nutrient consumption, or PA levels.

In another study by Woeber et al. [106], an experimental group followed a diet poor in processed carbohydrates and animal proteins and rich in omega-3 fatty acids, vitamins C and D, antioxidants, plant nitrates, and fiber for 4 weeks, while a control group maintained its usual diet. Both groups suspended interdental cleaning. The results showed that the experimental group had a significant reduction in gingival bleeding, an increase in vitamin D levels, and weight loss, with no changes observed in PI or serological inflammatory markers.

A cross-sectional study conducted by Marruganti et al. demonstrated that individuals with high adherence to the MD exhibited a markedly lower prevalence of advanced periodontitis (Stages III/IV), with only 29.66% affected, in contrast to 70.34% among those with low dietary adherence. Additionally, several clinical periodontal indicators—such as the percentage of PPD greater than 4 mm, the proportion of PPDs between 5 and 6 mm, presence of furcation involvement, tooth mobility, number of bleeding sites, and the number of teeth lost due to periodontal disease—were all significantly more severe in participants with poor adherence to the MD [107].

In a more recent study by the same research group [108], it was observed that low MD adherence and smoking were linked to worse PPD, REC, and CAL changes after periodontal therapy. Low PA, poor sleep, and excessive alcohol consumption were associated with higher odds of BoP-positive sites at 3 months. Individuals with unhealthy lifestyles (low MD, low PA, moderate/high PSS, poor sleep) were less likely to reach periodontal healing and showed more residual PPD ≥ 6 mm (OR = 0.85).

This finding contrasts with a study by Iwasaki et al. [109], which found no significant association between MD and periodontitis. However, the authors noted that olive oil consumption, a key component of the MD, was significantly inversely associated with periodontitis (adjusted OR = 0.55; 95% CI, 0.32–0.96). The inconsistency with the findings of Marruganti et al. may be explained by variations in the demographic characteristics of the study populations. The Moroccan study focused on university students with a mean age of approximately 20 years, whereas Marruganti et al. investigated individuals attending a periodontal clinic at a public university hospital in Italy, whose average age was 53. The younger cohort in the Moroccan study likely exhibited more favorable oral hygiene practices, reducing their susceptibility to periodontitis.

Furthermore, a recent systematic review and meta-analysis by Aalizadeh et al. [110] examined the potential relationship between adherence to the MD and periodontitis. While certain individual studies suggested a possible link, the overall pooled analysis did not reveal a statistically significant association, reporting an OR of 0.77 (95% CI: 0.58–1.03; *p* = 0.08).

### 3.3. Plant-Based Diet 

Nowadays, plant-based diets (PBDs) have gained traction in industrialized nations with a relatively high socio-economic status [111]. India has the largest proportion of vegetarians, with around 40% of its population adhering to a strict vegetarian diet. This is primarily due to cultural factors and the fact that many individuals are raised in vegetarian households. In contrast, in Western societies, the percentage of vegetarians ranges from 1% to 9%, where adopting a vegetarian diet is often a personal lifestyle decision [112]. Several studies in the scientific literature have demonstrated that a PBD has a protective effect against various chronic inflammatory diseases [113].

Adherence to vegetarian and vegan dietary patterns has been linked to reductions in BMI, TC, LDL cholesterol, glucose levels, and serum C-reactive protein (CRP), along with a reduced risk of cardiovascular diseases, obesity, type 2 diabetes, and certain cancers. Although vegan diets are often associated with decreased intake of specific micronutrients—such as vitamins B2, B12, and D, as well as iodine, zinc, calcium, and selenium—nutritional adequacy can be achieved through carefully structured diets, the use of fortified foods, and appropriate sun exposure for vitamin D synthesis. While the general health benefits of PBDs are well-supported in the literature, their impact on oral health remains controversial. A recent systematic review and meta-analysis by Azzola et al. [114] found no statistically significant differences in PPD, gingival recession, or CAL between individuals following vegan or vegetarian diets and those consuming omnivorous diets. Interestingly, the analysis did reveal a higher prevalence of tooth mobility among omnivores compared to plant-based diet followers. However, the authors cautioned that these findings should be interpreted carefully, as the duration of adherence to vegan or vegetarian diets varied considerably across the included studies.

Li et al. [115] evaluated the quantitative and qualitative influence of PDs on periodontitis using the plant-based diet index (PDI) [116,117]. They compared a healthful plant-based diet (hPDI), rich in whole foods, with an unhealthy plant-based diet rich in plant-derived processed foods (uPDI). Results showed an inverse association between the risk of developing periodontitis and hPDI, whereas uPDI increased the risk. Additionally, the study highlighted that patients exposed to an hPDI had higher serum antibody levels against periodontal pathogenic bacteria compared to the other group. These findings underscore how healthy diets, such as those rich in fruits, vegetables, whole grains, lean proteins, and fat-restricted foods, may positively affect the pathogenesis of periodontitis by modulating the host’s immune function. A systematic review of the literature by Woelber et al. [118] revealed that dietary modification, including a PD and supplementation with micronutrients, ω-3 fatty acids, green/oolong tea, polyphenols, and flavonoids, could be beneficial for NSPT, positively affecting periodontal parameters such as PPD, BoP, and CAL.

### 3.4. Ketogenic Diet 

Another diet gaining attention is the ketogenic diet (KD), characterized by low carbohydrates (less than 10%), moderate protein, and high fat [119]. When carbohydrates are reduced to less than 50 g daily, glycogen stores are depleted, and the body produces ketone bodies as an alternative energy source. It has been observed that, while this diet improves glycemic control in diabetic patients [120] and reduces cardiovascular risk, reduced intake of fiber and fruits, vegetables, and whole grains may induce constipation [119], hepatic steatosis, and hyperproteinemia. Few studies address the link between the KD and periodontitis. Rajaram et al. [121] observed that patients exposed for four weeks to a low-carbohydrate diet rich in ω-3 fatty acids, ascorbic acid, antioxidants, and fiber showed improvements in gingival inflammatory indices (BoP and GI), despite no difference in the PI between the test and control groups. A pilot study by Woelber et al. [122] evaluated 20 volunteers in good general health exposed to a KD for six weeks and found no improvements regarding clinical parameters related to periodontal inflammation, except for a lower PI compared to baseline. However, there was a significant reduction in BMI and weight loss. The regression analysis showed that fat mass and BMI were significantly positively correlated with periodontal inflammation, while HDL, fiber, and protein intake were negatively correlated.

However, the limitation of the KD is its high saturated fat content, which is associated with increased LDL cholesterol and systemic inflammation. The connection between periodontitis and high serum cholesterol levels is well-documented [123,124,125]. A recent systematic review of the literature by Taher et al. [126] concluded that current literature indicates a potential link between the KD and periodontitis, although the findings remain inconclusive. It can be suggested that this relationship may be beneficial, given the KD’s demonstrated anti-inflammatory effects, which reduce inflammatory markers commonly associated with various diseases, including periodontitis. Given the high prevalence of periodontitis and the promising therapeutic potential of the KD, this connection warrants further investigation. However, it should not be overlooked that the KD presents adverse short- to medium-term effects, such as cognitive decline, hepatic steatosis, hypoproteinemia, hypocitraturia, hypercalciuria, kidney stones, and vitamin and mineral deficiencies.

### 3.5. Stone-Age Diet

Another diet associated with oral health is the Stone-age diet (SD). It is well-known that this type of nutrition, a plant-food-based diet with a wide variety of fruits, nuts, and vegetables and minimal to no grains, dairy products, or sugar, offers significant benefits in reducing cardiovascular risk, insulin resistance, better glycemic control, and lowering blood pressure [127,128]. To the best of our knowledge, the first published study on the relationship between the SD and oral health was conducted by Baumgartner et al. [129], where a group of 10 volunteers were exposed to an environment replicating the SD period, circa 4000–3500 BC (housing, clothing, tools, and food). The diet was limited to whole grains such as barley, wheat, and spelt, some salt, herbs, honey, milk, and meat from domesticated animals (goats and chickens), without access to refined sugars. Since the diet was incomplete, participants were required to forage for food from nature, including berries, edible plants, and fish without nets. After four weeks, the results showed a reduction in the sites with BoP (from 34.8% to 12.6%; *p* < 0.001), while the PI increased from 0.68 to 1.47 (*p* < 0.001). The authors concluded that despite abstaining from oral hygiene practices and diet restrictions, there was no increase in gingival inflammation.

## 4. The Oral–Gut Microbiota Axis: A Bidirectional Interface Influenced by Diet

### 4.1. Oral Microbiota as a Determinant of Gut Microbial Composition

Recent metagenomic investigations have elucidated the influential role of the oral microbiota in shaping gastrointestinal microbial ecology [130,131,132]. In individuals with periodontal disease, the oral cavity functions as a reservoir of pathobionts capable of translocating to and disrupting distal mucosal environments. Translocation occurs via two primary mechanisms: (1) a hematogenous route, wherein pathogens such as *Fusobacterium nucleatum* and *Porphyromonas gingivalis* infiltrate systemic circulation through inflamed or ulcerated periodontal tissues, and (2) an enteral route, in which viable oral microbes are continuously swallowed and may colonize the gut under conditions such as hypochlorhydria or immunosuppression. Epidemiological data suggest that up to 30% of the gut microbiome may derive from oral taxa in individuals with inflammatory or metabolic disorders, including obesity, diabetes, and inflammatory bowel disease [130,133,134].

Oral dysbiosis, particularly in the context of periodontitis, leads to elevated salivary levels of proteolytic and inflammatory bacterial species. *P. gingivalis* has been shown to compromise intestinal epithelial barrier function through the downregulation of tight junction proteins such as occludin and ZO-1, resulting in increased intestinal permeability and systemic endotoxemia [134,135,136]. This process is further exacerbated by a shift in mucosal immunity—characterized by Th17 polarization, activation of NF-κB pathways, and depletion of SCFA-producing genera such as *Faecalibacterium prausnitzii*. Collectively, these alterations predispose the host to gastrointestinal inflammation, colorectal tumorigenesis, and metabolic dysfunction [131,132]. 

Clinical studies confirm the systemic implications of oral microbial imbalances. Kamer et al. [137] reported that individuals with moderate to severe periodontitis exhibit distinct gut microbial signatures, including reduced abundance of *Bifidobacterium* and *Eubacterium rectale*, alongside increased fecal calprotectin and zonulin levels—biomarkers of intestinal inflammation and barrier dysfunction. These changes were present even in the absence of clinically manifest gastrointestinal disease, indicating a subclinical pro-inflammatory state potentially originating from oral dysbiosis.

Moreover, a clarifying and insightful longitudinal study by Baima et al. [138] delineated the microbial interplay between the oral cavity and the gut in individuals with advanced periodontitis, aiming to evaluate how non-surgical periodontal therapy may influence these microbial ecosystems over time. Initial comparisons revealed a significant dissimilarity in gut microbial diversity (β-diversity) between patients with periodontitis and healthy controls (<0.001). At baseline, the fecal microbiota of periodontitis patients exhibited elevated levels of *Bacteroides*, *Faecalibacterium*, *Fusobacterium*, and members of the *Lachnospiraceae spp*., whereas Lactobacillus was more abundant in the healthy cohort. Following treatment, a marked remodeling of both the oral and gut microbiota was observed (*p* < 0.001), with post-treatment gut microbial profiles shifting toward those of the control group.

Notably, NSPT resulted in a concomitant reduction of both oral pathogens and gut-associated taxa—including *Bacteroides*, *Lachnoclostridium*, *Lachnospiraceae*, *Oscillospiraceae*, and *Ruminococcaceae*—bringing their relative abundances in line with levels typically found in healthy individuals. These findings underscore the existence of a bidirectional relationship between periodontal health and intestinal microbial homeostasis. Importantly, therapeutic intervention targeting periodontal inflammation appears not only to reestablish oral microbial balance but also to exert a corrective influence on gut microbiota composition. 

### 4.2. Dietary Modulation of the Oral–Gut Microbiota Axis

Diet plays a crucial regulatory role in shaping the oral–gut microbial continuum by modulating microbial substrates, host immune responses, and epithelial integrity.

The WD pattern, characterized by a high intake of saturated fats, refined sugars, and ultra-processed foods, is consistently associated with oral and intestinal dysbiosis.

It promotes expansion of Proteobacteria (e.g., *Escherichia, Shigella, Klebsiella, Bilophila wadsworthia*), known producers of pro-inflammatory lipopolysaccharide (LPS). This is accompanied by reductions in beneficial taxa such as Bacteroidetes, Lactobacillus, Roseburia, and F. prausnitzii, leading to Short-Chain Fatty Acid (SCFA) depletion, downregulation of tight junction proteins (e.g., ZO-1, occludin), increased intestinal permeability, and chronic systemic inflammation. WD also stimulates TLR4-mediated inflammatory signaling and enhances TMAO production from dietary choline and carnitine, contributing to cardiometabolic risk [139,140]. 

In contrast, MD promotes eubiosis by increasing microbial diversity and fostering the proliferation of beneficial commensals such as *F. prausnitzii and E. rectale* (butyrate-producing Firmicutes), *Bifidobacterium* spp. (linked to carbohydrate metabolism and immune regulation), and *Bacteroides* spp. (crucial for polysaccharide degradation) [140,141].

Simultaneously, MD reduces pathogenic *Proteobacteria* and pro-inflammatory *Firmicutes* (e.g., *Blautia*). Polyphenols from olive oil and red wine are metabolized into phenolic acids that inhibit NF-κB and reduce TNF-α/IL-6 while upregulating IL-10. SCFAs generated via saccharolytic fermentation enhance tight junction expression (occludin, claudins), protecting against LPS translocation and systemic inflammation. PUFAs in fish and nuts promote *Akkermansia muciniphila*, supporting mucin degradation, epithelial integrity, and lipid metabolism [141,142].

Intake of fermented foods, which are the result of microbial-driven biotransformation processes in which endogenous or inoculated microorganisms metabolize carbohydrates and other nutrients, leading to the production of metabolites—such as organic acids, alcohols, gases, and bioactive peptides—act as potent microbiota modulators through [140,143,144]:

(a) Transient Colonization: Delivering viable bacteria (e.g., Lactobacillus, Leuconostoc, Pediococcus, Bifidobacterium) that temporarily interact with the host gut ecosystem.
(b) Substrate Liberation: Enhancing availability of microbiota-accessible carbohydrates (MACs) and producing SCFAs through fermentation.
(c) Immunomodulation: Influencing TLR signaling, NF-κB inhibition, and regulatory T-cell activation via bioactive metabolites such as exopolysaccharides and peptides.

In a recent review by Leeuwendaal et al. [143], the authors concluded that fermented foods represent a promising avenue for modulating the gut microbiota through two primary mechanisms: (i) by supplying substrates that selectively enhance or suppress the growth of specific microbial taxa within the intestinal ecosystem and (ii) by introducing exogenous microorganisms—originating from the food microbiome—that may either transiently colonize the gastrointestinal tract or interact functionally with the existing gut microbiota.

Despite their theoretical potential, empirical evidence directly examining the influence of fermented food intake on gut microbial composition and function remains limited. Nonetheless, several microbial metabolites generated during fermentation—such as polyphenolic derivatives and SCFAs—have been identified as possessing biologically beneficial properties upon ingestion.

As for the KD, it elicits a biphasic response to gut microbiota. During the initial phase, there is a reduction in SCFA-producing bacteria such as *Bifidobacterium* and *Eubacterium rectale* accompanied by decreased α-diversity. However, with long-term adherence, KD leads to a decline in pro-inflammatory taxa such as Desulfovibrio and Turicibacter, which are associated with mucosal inflammation. This is accompanied by suppression of Th17 cells and pro-inflammatory cytokines through microbiota-mediated inhibition of dendritic cell maturation and IL-6 signaling. Additionally, KD promotes microbial GABA synthesis and modulation of the tryptophan–serotonin pathway—mechanisms implicated in gut–brain axis regulation [140,145,146].

In conclusion, the oral–gut microbiota axis represents a vital yet underrecognized interface through which host–microbe interactions influence systemic health. Periodontal dysbiosis, via microbial translocation and immune activation, may initiate or exacerbate intestinal inflammation and metabolic dysregulation. Diet emerges as a central modulator of this axis, capable of either disrupting (e.g., WD) or restoring (e.g., MD, fermented foods) host–microbiota equilibrium. These findings emphasize the need for integrative dietary and microbiome-targeted strategies in the prevention and management of chronic inflammatory and metabolic diseases.

## 5. Obesity, Physical Activity, and Periodontal Health

### 5.1. Obesity and Periodontitis

Overweight and obesity are characterized by an excessive accumulation of body fat that can negatively affect health. An individual is classified as overweight if their BMI is ≥25 and obese if their BMI is ≥30 kg/m^2^ [147]. The global prevalence of overweight and obesity has risen significantly in recent decades, leading to growing concerns about their health and socioeconomic consequences. 

Obesity is often associated with a state of chronic low-grade inflammation and has been linked to an increased risk of developing major chronic conditions, such as cardiovascular diseases and diabetes, and may also contribute to the development of periodontitis [148]. 

In fact, obesity can impact serum concentrations of pro-inflammatory mediators [such as IL-6, CRP, TNF-α, resistin, and leptin] and anti-inflammatory mediators (Adiponectin and IL-10) in individuals with periodontitis. Conversely, periodontitis can also modify the levels of these mediators in obese patients, further worsening their inflammatory profiles [149,150,151].

An interesting experimental study on an animal model by Virto et al. [152] evaluated the periodontal and systemic effect of the relationship between obesity and overweight with periodontitis. The results demonstrated that clinical parameters, including PPD and GI, were notably higher in the induced periodontitis rat groups compared to the control groups. Specifically, the high-fat diet Perio group (HFD-Perio group) showed a significantly greater PD than the rats with induced periodontitis but with a controlled diet. Lipid profiles, cytokines, and adipocytokines were also substantially elevated in the rats with a high-fat diet and affected by periodontitis when compared to the other groups, as well as for glucose and markers of liver damage compared to other groups. The study concluded that obesity and periodontitis exhibit a comorbid effect, contributing to systemic inflammation and metabolic disruptions, such as elevated glucose levels, dyslipidemia, and liver damage. 

Different research confirmed the existence of a strong association between obesity and periodontitis (OR 1.2–4.5) [153]; specifically, obesity independently enhances the risk of periodontitis by two to three times, irrespective of traditional risk factors like age, gender, and smoking [154,155]. 

Several studies have examined the relationship between obesity and the severity of periodontitis, though this association is not straightforward [156]. Although obesity has been associated with an increased prevalence of mild to moderate periodontitis, there is insufficient evidence to establish a significant link between obesity and severe forms of periodontal disease. After adjusting for confounding factors such as smoking, only a weak dose–response relationship was observed between BMI and the presence of deeper periodontal pockets [157]. A cross-sectional study reported an inverse association between BMI and CAL, suggesting that obesity may act as a precursor to gingival inflammation rather than being directly linked to the advanced breakdown of periodontal tissues [158]. This finding highlights the potential influence of obesity-related metabolic alterations on gingival health during the initial phases of disease development. Furthermore, the results point to the possibility that early inflammatory disruptions in tissue vascularization may mediate the observed relationship between obesity and periodontitis [159]. Moreover, the link between obesity and periodontal disease may intensify with higher levels of obesity, suggesting that greater obesity severity exacerbates the inflammatory effects on periodontal tissues. Additionally, insufficient interdental cleaning may exacerbate gingival bleeding in obese individuals [159]. Thus, obesity potentially results in a persistent pro-inflammatory state that induces a modification in the micro-environment of the periodontal sites, favoring the growth and complexity of oral microflora [160]. A very important study by Azuma et al. [161] was conducted on an animal model aiming to examine the impact of exercise training on serum oxidative stress, assessed as serum reactive oxidative metabolites (ROM) and 8-OHdG, and gingival oxidative stress in obese rats subjected to a high-fat diet. The results demonstrated that obese rats on a high-fat diet without exercise training exhibited significantly higher serum ROM levels compared to the control groups, i.e., rats fed a regular diet with and without physical exercise. This condition also resulted in elevated 8-OHdG levels in gingival tissues compared to the control group at 8 weeks. Furthermore, the real interesting aspect was that obese rats on a high-fat diet with exercise training displayed lower serum ROM levels and gingival 8-OHdG levels compared to those without exercise training. A similar concept was confirmed in a pilot study by Park et al. [162] conducted on a human model, which demonstrated that all obese volunteer patients successfully reduced their weight during a 4-week weight control program. Key findings included significant reductions in BMI, waist-to-hip ratio (WHR), WC, and serum LDL levels, while HDL levels remained unchanged, and triglycerides increased unexpectedly. No significant changes were observed in serum CRP, an indicator of systemic inflammation. Markers of periodontal health, such as the GI and BoP, showed no significant changes, likely due to high variability. However, inflammatory biomarkers in gingival GCF, including IL-1β, MMP-8, and MMP-9, significantly decreased by 60.2%, 58.6%, and 35.8%, respectively, in the obese group. In contrast, no significant changes were observed in these biomarkers in the normal weight group [162]. 

### 5.2. Relationship Between NSPT and Obesity

A robust correlation exists between the clinical success of NSPT and obesity, a relationship that has been confirmed in both human and animal models, as demonstrated in a notable study by Pereira et al. [163]. The authors examined the effects of periodontitis and periodontal therapy on systemic inflammation and metabolic parameters in both obese and non-obese rat models. Their findings revealed that periodontitis led to elevated serum CRP levels in obese rats, underscoring the compounded impact of periodontitis on the systemic inflammation associated with obesity. In contrast, the treatment of induced periodontitis had only a limited effect on overall systemic inflammation. Furthermore, periodontal treatment in rats was associated with reduced total cholesterol (TC) levels, suggesting that effective management of periodontitis may have beneficial effects on metabolic control.

In a systematic review and metanalysis from 10 years ago by Papageorgiou et al. [164], it was highlighted that periodontal therapy in systemically healthy overweight or obese patients resulted in a more significant reduction in TNF-α levels and a greater decrease in HbA1c levels compared to systemically healthy normal-weight individuals. In contrast to diabetic normal-weight patients, periodontal treatment in diabetic overweight/obese patients led to an increase in adiponectin levels (anti-inflammatory mediator) and a decrease in leptin levels (pro-inflammatory mediator). The authors concluded that the available evidence is limited due to inconsistencies, imprecision, and a lack of studies. This was confirmed in another recent systematic review by Zhang et al. [165] that evaluated the effect of NSPT on inflammatory biomarkers in patients with or without obesity. At baseline, levels of pro-inflammatory biomarkers—including serum IL-6, TNF-α in both serum and GCF, CRP/high sensitivity-CRP, and resistin—were significantly elevated in obese individuals compared to those of normal weight. Following non-surgical periodontal NSPT, a shift toward an anti-inflammatory profile was observed, characterized by decreased concentrations of pro-inflammatory cytokines such as IL-6 and increased levels of anti-inflammatory markers, including adiponectin. However, TNF-α, which is more strongly linked to obesity severity and insulin resistance, showed no significant change three months post-treatment [166]. The results of the meta-analysis indicate that NSPT might be more effective in lowering overall serum levels of CRP/hs-CRP at the 3-month follow-up, with no significant difference observed between individuals with and without obesity. However, at baseline, serum levels of CRP/hs-CRP were notably higher in patients with obesity compared to those without obesity, indicating a high pro-inflammatory status. NSPT led to improvements in clinical parameters for both groups at the 3 and 6-month evaluations. However, patients without obesity showed a lower mean PPD at 6 months post-treatment as well as a more significant reduction in PPD from baseline to 6 months in the full-mouth analysis and in sites with initially deeper PPD. Leptin serum levels were consistently higher in obese patients compared to non-obese patients at all time points. No significant changes in leptin and adiponectin serum levels were observed in either group following therapy.

Several studies have also shown that patients with obesity presented a worse response to periodontal treatment due to high systemic inflammation. For example, a study by Gonçalves et al. [167] compared the effect of SRP on clinical outcomes and circulating leptin and adiponectin levels in patients affected by periodontitis with and without obesity. The results showed that SRP improved periodontal clinical parameters for both groups at 3 and 6 months, but patients without obesity showed a lower mean PPD at 6 months post-treatment as well as a more significant reduction in PPD from baseline to 6 months in the full-mouth analysis and in sites with initially deeper PPD compared to obese patients. Furthermore, leptin serum levels were consistently higher in obese patients compared to non-obese patients at all time points. However, no significant changes in leptin and adiponectin serum levels were observed in either group following therapy. 

More recent research by Zuza et al. [168] assessed the recurrence of periodontitis over a two-year follow-up period in individuals with obesity and those of normal weight, all of whom had undergone SRP. Both groups demonstrated comparable periodontal outcomes, with a generally low rate of periodontitis recurrence. Nevertheless, obesity was linked to heightened inflammatory responses in the GCF, marked by elevated concentrations of IL-6, TNF-α, and IL-1β. These pro-inflammatory cytokines may act as predictive markers for an increased risk of periodontal relapse, particularly in the context of suboptimal plaque control. Similar results were found by Kaye et al. [169], who concluded that obese and overweight individuals were more prone to undergo SRP or surgical interventions compared to those with normal weight, independent of initial disease severity. Furthermore, obese and overweight patients exhibited treatment intensity scores that were 40% and 24% higher, respectively, than those of normal-weight individuals. On the other hand, there are studies that have evaluated the influence of combining SRP with a healthier dietary regimen in obese patients, showing that modifying the diet leads to a better response to treatment. Martinez-Herrera et al. [170] evaluated the impact of weight loss on enhancing the response of obese individuals to NSTP, showing that the obese patient that follow a healthy dietary exhibited significantly greater reductions in mean probing depth and in the percentage of sites with a PPD of 4–5 mm. Furthermore, complement component 3 (C3) and TNFα decreased in the dietary group post-intervention. These findings suggest that weight loss through dietary intervention may reduce systemic inflammation, potentially improving periodontal treatment outcomes. 

Analogue results were confirmed in a pilot study by Lakkis et al. [171] in which a significant weight lost after bariatric surgery in obese patients affected by periodontitis and treated with SRP showed significantly greater improvements in PPD, CAL, BoP, and GI compared to patients not exposed to surgery. The authors concluded that weight loss in obese patients ensures a better response to periodontal therapy, likely due to the reduction of systemic inflammation.

### 5.3. Exercise and Systemic Inflammation: Biological Implications

Inflammation is commonly characterized by redness, fever, swelling, pain, and functional impairment, accompanied by leukocyte infiltration and the production of pro-inflammatory cytokines. These inflammatory responses act as protective mechanisms against external harmful stimuli, such as microbial infections [172,173]. Cytokines, produced by various cells within tissues, play a pivotal role in regulating inflammatory responses, even at minimal concentrations, through autocrine or paracrine signaling pathways. The key cytokines implicated in the early phases of acute inflammation include TNF-α, IL-1β, and IL-6. During a typical inflammatory response, TNF-α is the first to be systemically released, reaching its peak within hours after the onset, subsequently followed by the production of IL-1β and then IL-6 [174]. These pro-inflammatory cytokines drive subsequent acute inflammatory responses, such as leukocytosis (neutrophilia), through the induction of granulocyte colony-stimulating factor (G-CSF) and chemokines, including IL-8 and monocyte chemotactic protein (MCP)-1 [175,176,177]. However, prolonged exposure to these inflammatory mediators can contribute to the development of chronic metabolic diseases, including diabetes, cardiovascular diseases, chronic kidney disease, and cancer [178].

Exercise is a purposeful, structured PA aimed at maintaining physical fitness and overall health [179]. In recent years, exercise has been recognized as an anti-inflammatory intervention [180]. Regular PA is therefore regarded as a natural defense against chronic inflammatory diseases, partly by promoting the release of anti-inflammatory cytokines into the bloodstream [181,182]. In fact, numerous studies have shown that endurance exercise lasting several hours leads to increased levels of IL-6, IL-10, IL-1, and IL-8 [181,183,184,185]. However, during or after short-duration, high-intensity exercise [180,182], or eccentric contraction-based exercise [186], the cytokine response is minimal. These findings suggest that the cytokine response is more closely related to the intensity and duration of exercise (physiological load/stress) rather than to muscle damage induced by exercise [180].

IL-6, a pivotal cytokine, exhibits a marked increase following prolonged endurance exercise, contributing to enhanced lipid mobilization—particularly the release of free fatty acids—which supports improved endurance capacity [187]. Beyond its metabolic role, IL-6 facilitates the recruitment and activation of neutrophils and stimulates the production of anti-inflammatory cytokines, including IL-1ra and IL-10 [188,189]. IL-10 is recognized for its potent immunosuppressive properties, while IL-1ra functions as a competitive inhibitor of IL-1 by occupying its receptor without triggering downstream signaling. Physical activity also induces the secretion of IL-8, an essential chemokine for neutrophil chemotaxis and activation, with particularly elevated levels observed during extended, high-intensity efforts such as marathon running [187,188,189,190,191]. Notably, even brief bouts of intense exercise—such as eccentric or exhaustive activity lasting approximately 10 minutes—can significantly elevate plasma IL-8 levels [189]. 

### 5.4. Physical Activity and Periodontal Health

Evidence suggests a potential relation between PA and oral health. A recent study by de Olivera et al. [192] on an animal model evaluated the effects of a moderate-intensity physical training protocol on ABL in rats with ligature-induced periodontitis; in addition, the authors evaluated inflammatory cytokines such as IL-1β, IL-6, TNF-α, and IL-10; C-reactive protein; lipid peroxidation (LPO); and reduced glutathione using histological and microtomographic evaluation. The results showed that PA significantly reduced IL-1β, IL-6, TNF-α, CRP, and LPO levels and increased IL-10 levels in the periodontitis group; moreover, the histological analysis showed reduced inflammatory infiltrate and less fiber degradation. Furthermore, the periodontitis + training group showed less vertical bone loss and a higher bone volume/trabecular volume ratio. These findings suggest that moderate-intensity physical exercise may help reduce inflammation and damage in periodontitis.

The World Health Organization recommends moderate exercise, with 150 min per week associated with a lower prevalence of lifestyle-related diseases, including periodontitis [179,193]. A systematic review and meta-analysis identified a limited number of studies linking PA to reduced periodontal disease prevalence, with more frequent activity correlating with a lower occurrence of periodontitis [194]. Additionally, evidence highlights associations between obesity, physical fitness, and periodontitis [195,196,197]. The 2020 guidelines on PA and sedentary behavior [198] emphasized that even small amounts of physical activity and a reduction in sedentary behavior are more beneficial than none for all populations. It is recommended that individuals begin with low levels of activity and gradually increase the frequency, intensity, and duration over time. The relationship between PA and inflammatory conditions, such as periodontitis, is thought to be mediated by the modulation of inflammation. Almohamad et al. found that patients engaging in any level of PA (greater than 0 min/week) had lower rates of periodontal disease compared to those with no PA (0 min/week). Additionally, individuals with low sedentary behavior had lower rates of periodontal disease than those with high sedentary behavior; in particular, adjusted multivariable regression models revealed that increased sedentary behavior (over 7.5 h per day) was associated with higher odds of periodontal disease (OR = 1.17; 95% CI = 1.00–1.36; *p* = 0.045) [199].

However, a study by Rui Pu et al. [200] revealed that periodontitis prevalence was significantly correlated with activity categories. After adjusting for age, sex, race, BMI, diabetes, smoking status, alcohol use, and flossing frequency, significant associations between activity levels and periodontitis remained. Both work and recreational activities showed contrasting effects. Moderate and vigorous work PA increased the odds of periodontitis by 22% and 40%, respectively, while moderate and vigorous recreational activity reduced the odds by 19% and 45%. Periodontitis prevalence was also higher in individuals with diabetes, smokers, heavy drinkers, and those with low flossing frequency. Participants engaged in work PA had higher odds of periodontitis, while moderate or vigorous recreational activity was associated with a lower prevalence of periodontitis.

This narrative review aims to synthesize current knowledge on the interrelationship between dietary patterns, physical activity, and periodontal health. Nonetheless, several methodological limitations should be acknowledged. 

Firstly, it lacks a systematic and transparent search strategy, which may result in selection bias. The inclusion of studies was not governed by predefined eligibility criteria, and as such, may reflect the authors’ subjective judgment rather than a comprehensive or balanced representation of the existing evidence. However, to enhance the credibility and scientific relevance of the discussion, the authors prioritized references from peer-reviewed articles published in high-impact journals in the field of periodontology and related disciplines. 

Secondly, the absence of a structured quality assessment of the included studies means that studies with a high risk of bias or limited validity are given equal interpretative weight alongside more robust investigations.

Thirdly, due to the qualitative nature of narrative reviews, no quantitative synthesis (such as a meta-analysis) was conducted. This restricts the ability to evaluate the magnitude, consistency, or statistical significance of associations across different studies. As a result, conclusions drawn from this review remain largely descriptive and should not be interpreted as establishing causality.

Lastly, although this review provides a broad overview of the potential links between nutrition, physical activity, and periodontal disease, the lack of a standardized methodology inherent to the narrative format limits the generalizability and applicability of its findings. 

## 6. Conclusions

The influence of diet and PA on periodontal health is becoming increasingly recognized as a critical component in the prevention and management of periodontal diseases. Micronutrients such as vitamin C, vitamin D, omega-3 fatty acids, calcium, iron, zinc, and magnesium play key roles in supporting periodontal tissues and modulating the inflammatory response. 

An improper diet predisposes individuals to systemic inflammation, increasing the risk of developing obesity and all associated complications. It has been observed that not only do obese patients develop periodontitis more frequently, but they may also exhibit a poorer response to periodontal therapy. 

Regular PA reduces systemic inflammation, enhances immune function, and increases antioxidant defenses, all of which contribute to improved periodontal health. The combined effects of a healthy diet and regular PA may represent a benefit for the prevention and management of periodontal diseases, underscoring the importance of these modifiable lifestyle factors in periodontal care.

However, future research employing systematic approaches, along with well-designed longitudinal and interventional studies, is warranted to clarify these associations and support the development of evidence-based clinical guidelines.

## Abbreviations

PA	Physical activity
ROS	Reactive oxygen species
SVCT2	Sodium-Dependent Vitamin C Transporter 2
DHA	Dehydroascorbate
NF-κB	Nuclear Factor kappa-light-chain-enhancer of activated B cells
CAL	Clinical attachment loss
CRP	C-reactive protein
TOSC	Total oxyradical scavenging capacity
GCF	Gingival crevicular fluid
8-OHdG	8-hydroxy-2’-deoxyguanosine
MMP	Matrix Metalloproteinase
ABL	Alveolar bone loss
TNF-α	Tumor Necrosis Factor-alpha
25(OH)D	25-hydroxyvitamin D
1,25(OH)_2_D_3_	1,25-dihydroxyvitamin D₃
NSPT	Non-surgical periodontal therapy
SRP	Scaling and Root Planing
PPD	Probing depth
BoP	Bleeding on probing
VDR	Vitamin D receptor
TLR	Toll-like receptor
SNP	Single nucleotide polymorphism
DBP	Vitamin D-binding protein
MAF	Macrophage activating factor
PUFAs	Polyunsaturated fatty acids
EPA	Eicosapentaenoic acid
DHA	Docosahexaenoic acid
SPMs	Specialized pro-resolving mediators
GI	Gingival Index
BI	Bleeding Index
MD	Mediterranean diet
Zn^2^⁺	Zinc Ion
SOD	Superoxide dismutase
OR	Odds ratio
CI	Confidence interval
WD	Western diet
BW	Body weight
BMI	Body Mass Index
WC	Waist circumference
TMAO	Trimethylamine N-oxide
TC	Total cholesterol
HDL	High-Density Lipoprotein
oxLDL	Oxidized Low-Density Lipoprotein
LDL	Low-Density Lipoprotein
SFAs	Saturated fatty acids
PI	Plaque Index
PISA	Periodontal Inflamed Surface Area
PBD	Plant-based diet
hPDI	Healthful Plant-Based Diet Index
uPDI	Unhealthful Plant-Based Diet Index
KD	Ketogenic diet
SD	Stone-age diet
LPS	Lipopolysaccharide
SCFAs	Short-Chain Fatty Acids
MACs	Microbiota-accessible carbohydrates
GABA	Gamma-aminobutyric acid
WHR	Waist-to-hip ratio
MCP-1	Monocyte Chemoattractant Protein-1
G-CSF	Granulocyte colony-stimulating factor
IL-1ra	Interleukin-1 Receptor Antagonist
LPO	Lipid peroxidation
